# The new final Clinical Skills examination in human medicine in Switzerland: Essential steps of exam development, implementation and evaluation, and central insights from the perspective of the national Working Group

**DOI:** 10.3205/zma000982

**Published:** 2015-10-15

**Authors:** Christoph Berendonk, Christian Schirlo, Gianmarco Balestra, Raphael Bonvin, Sabine Feller, Philippe Huber, Ernst Jünger, Matteo Monti, Kai Schnabel, Christine Beyeler, Sissel Guttormsen, Sören Huwendiek

**Affiliations:** 1 University of Bern, Medical Faculty, Institute of Medical Education, Bern, Switzerland; 2 University of Zurich, Medical Faculty, Zurich, Switzerland; 3 University of Basel, Medical Faculty, Basel, Switzerland; 4 University of Lausanne, Medical Faculty, Lausanne, Switzerland; 5 University of Geneve, Medical Faculty, Geneve, Switzerland

**Keywords:** national final examination, licensing examination, summative assessment, OSCE, action research

## Abstract

**Objective: **Since 2011, the new national final examination in human medicine has been implemented in Switzerland, with a structured clinical-practical part in the OSCE format. From the perspective of the national Working Group, the current article describes the essential steps in the development, implementation and evaluation of the Federal Licensing Examination Clinical Skills (FLE CS) as well as the applied quality assurance measures. Finally, central insights gained from the last years are presented.

**Methods: **Based on the principles of action research, the FLE CS is in a constant state of further development. On the foundation of systematically documented experiences from previous years, in the Working Group, unresolved questions are discussed and resulting solution approaches are substantiated (planning), implemented in the examination (implementation) and subsequently evaluated (reflection). The presented results are the product of this iterative procedure.

**Results: **The FLE CS is created by experts from all faculties and subject areas in a multistage process. The examination is administered in German and French on a decentralised basis and consists of twelve interdisciplinary stations per candidate. As important quality assurance measures, the national Review Board (content validation) and the meetings of the standardised patient trainers (standardisation) have proven worthwhile. The statistical analyses show good measurement reliability and support the construct validity of the examination. Among the central insights of the past years, it has been established that the consistent implementation of the principles of action research contributes to the successful further development of the examination.

**Conclusion: **The centrally coordinated, collaborative-iterative process, incorporating experts from all faculties, makes a fundamental contribution to the quality of the FLE CS. The processes and insights presented here can be useful for others planning a similar undertaking.

## Zusammenfassung

**Zielsetzung: **Seit 2011 wird in der Schweiz eine neue nationale Schlussprüfung in Humanmedizin mit einem strukturierten klinisch-praktischen Teil im OSCE Format durchgeführt. Der vorliegende Beitrag beschreibt, aus Sicht der nationalen Arbeitsgruppe, die essentiellen Schritte der Erstellung, Durchführung und Auswertung der Eidgenössischen Prüfung Clinical Skills (EP CS) sowie der jeweils eingesetzten Qualitätssicherungsmassnahmen. Schliesslich werden zentrale Erkenntnisse der vergangenen Jahre vorgestellt.

**Methodik: **Basierend auf den Prinzipien der Aktionsforschung wird die EP CS kontinuierlich weiter entwickelt. Auf der Grundlage systematisch dokumentierter Erfahrungen aus den Vorjahren werden in der Arbeitsgruppe anstehende Fragen diskutiert, daraus resultierende Lösungsansätze konkretisiert (Planung), in der Prüfung umgesetzt (Implementation) und anschliessend evaluiert (Reflexion). Die vorgestellten Ergebnisse sind das Produkt dieses iterativen Vorgehens.

**Ergebnisse:** Die EP CS wird durch Fachexperten aller Fakultäten und Fachrichtungen in einem mehrstufigen Prozess erstellt. Die Prüfung wird dezentral in Deutsch und Französisch durchgeführt und besteht aus zwölf interdisziplinären Stationen pro Kandidat. Als wichtige Qualitätssicherungsmassnahmen haben sich das nationale Review Board (inhaltliche Validierung) und die Treffen der Schauspielpatienten-Trainer (Standardisierung) erwiesen. Die statistischen Auswertungen zeigen eine gute Messzuverlässigkeit und weisen auf die Konstruktvalidität der Prüfung hin. Zu den zentralen Erkenntnissen der vergangenen Jahre gehört, dass die konsequente Umsetzung der Prinzipien der Aktionsforschung zur erfolgreichen Weiterentwicklung der Prüfung beiträgt.

**Schlussfolgerung: **Der zentral koordinierte, kollaborativ-iterative Prozess mit Einbindung von Fachexperten aus allen Fakultäten trägt wesentlich zur Qualität der EP CS bei. Die hier vorgestellten Prozesse und Erkenntnisse können für andere nützlich sein, welche Ähnliches vorhaben.

## Introduction

In 2011, the changeover from the previous State Examination to the new Federal Licensing Examination in human medicine came into effect. The impetus and legal basis for this new national final examination was the Federal Law on Medical Professions (MedBG) adopted in June 2006 [https://www.admin.ch/opc/de/official-compilation/2007/4031.pdf cited at 14.12.13]. The educational objectives laid down in the MedBG refer to the “Swiss Catalogue of Learning Objectives for Undergraduate Medical Training” (SCLO) [1], which is mandatory for all five Swiss medical faculties and which lists and specifies the required competencies in detail. In February 2007, a national steering group was set up by the Federal Office of Public Health, which was entrusted with the responsibility for the strategic orientation of the new Federal Licensing Examination (FLE) in human medicine. This supreme steering committee resolved that the future FLE in human medicine should be an interdisciplinary examination with two parts. The first part is a written examination with a Multiple Choice (MC) format. The second part (Clinical Skills Examination) consists of a structured clinical-practical examination with standardised patients (SP) and is based on the principle of the Objective Structured Clinical Examination (OSCE) [2]. 

The overarching development process of the entire FLE in human medicine, from the concept to the first implementation, including the legal framework, the discourse about the available scientific evidence on the theme of summative assessments, and the development of the cross-examination blueprints, is described in detail elsewhere and is not included in this article [3]. The current work is concerned with the development of the FLE Clinical Skills (CS) as it has been administered since 2011.

Structured clinical-practical examinations as part of licensing examinations at the end of undergraduate training are known from Canada [4] and the USA [5], and have been successfully conducted there for several years. Licensing examinations are also common in Europe, but a national structured clinical-practical examination constitutes a novelty in the European context. Although there is a wealth of literature concerned with the most diverse of aspects of an OSCE [6], barely any studies have been published which investigate the specific characteristics of developing national clinical-practical examinations. Therefore, the aim of the current article is to describe – from the perspective of the Working Group CS – essential steps for the development, implementation and evaluation of the examination, as well as quality assurance measures and central insights gained from the last three years.

## Methods

The national Working Group Clinical Skills (WG CS) is constituted of members of the five medical faculties (Basel, Bern, Geneva, Lausanne, Zurich) and the Institute of Medical Education of the University of Bern. All members have many years of practical experience with the OSCEs conducted at the individual faculties. The procedure chosen by the WG CS for the further concept development of the FLE CS is based on the methodological principles of action research [7]. Key elements of this method are the iterative development process (planning-implementation-reflection) and the active, democratic participation of all stakeholders [8]. The primary focus is not on an individual scientific method, but on mutually defined questions, with the aim of continuously improving a product or process (in this case the FLE CS). Characteristic for action research are scientific questions grounded in everyday life, which lead to the generation of knowledge that is of relevance and interest outside of the local context [9].

The active participation of the WG CS members is ensured by eight half-day sessions per year, with the role of chairperson rotating from session to session. The knowledge of the individual Working Group members regarding processes and pitfalls within OSCEs is of fundamental importance. In the phase of *planning*, the individual members contribute their own experiences; together, questions and goals are defined, possible problem areas are identified, and possible solutions are sought. In this phase, reference is made to the existing scientific evidence on the respective theme. Furthermore, in terms of corresponding questions, contact is sought with the National Board of Medical Examiners of the USA and the Medical Council of Canada, in order to benefit from their long-standing experience with national clinical-practical exams. Prior to the *implementation*, the strategy developed by the WG CS is submitted to the Swiss Board of Examiners for approval. In the case of uncertainties regarding the selected strategy, the concept is first of all piloted in a faculty-based OSCE, and is only then implemented in the FLE CS. In order to evaluate the implemented strategy, a multitude of different qualitative and quantitative methods is employed. In the phase of *reflection*, the experiences gathered and results obtained are discussed in the WG CS with regard to questions or goals. If there is a further need for action, the initial concept is revised and the cycle starts again from the beginning. Each step of the FLE CS goes through this three-stage process of planning-implementation-reflection. As it would be beyond the scope of the current article to describe the whole process for each step, only the results of these processes are presented here. 

## Results

The processes of exam development, implementation, evaluation and quality assurance as well as the central insights presented in the following cannot, in reality, be considered as separate entities. Rather, they are interwoven and mutually influence one another. This division is made here to facilitate understanding.

### Exam development

**Case development:** The initial step of case development consists in identifying a theme which is suitable for the Clinical Skills (CS) examination. The foundation for possible themes is the “problems as starting points” chapter of the Swiss Catalogue of Learning Objectives and the requirements of the blueprint. Once a theme has been determined, an experienced clinician, selected according to the case theme, writes a first case draft, which is evaluated and commented on in terms of formal criteria by methodological experts. This draft is then developed further in the framework of a workshop together with a second clinician (from a different faculty and subject area), as well as a CS coach (person with experience of the CS exam format). In these workshops, not only is the actual case content developed, but also the weighting of the individual assessment criteria is defined. This team approach ensures that in the case development, the necessary medical subject competence is present (clinician) and the exam-format-specific aspects are taken into account (CS coach). The practicability of the developed cases is tested in the workshop itself with standardised patients (SP). These test runs provide important hints, which often have a substantial influence on the further case development.

**Structure of a Clinical Skills station: **The history-taking and physical examination to be performed by the candidate, as well as the differential diagnoses and the further diagnostic and therapeutic procedures (so-called ASM criteria: anamnesis, status, management) are assessed by means of case-specific checklists. The communication between candidate and SP (so-called CC criteria), by contrast, is rated on a generic scale which comprises four aspects and is identical for all stations [10], [11].

**Review process: **In the next step, the cases are presented to the national Review Board. This consists of representatives of the five medical faculties and representatives from general medicine. The task of the Review Board is to evaluate the cases in terms of their correctness, relevance and complexity. Following a critical discussion, the Review Board specifies the corresponding need for amendment or approves the case for the further development process.

**Standardisation: **Thereafter, the cases are made available to the SP trainers. The SP trainers of all five faculties develop a uniform understanding of the cases in joint meetings. Each case is discussed, acted out and videotaped. The video documentation enables complex situations (e.g. presentation of the patient’s mental health) or the degree of a certain symptom (e.g. neurological deficit) to be precisely defined. Within these meetings, it is also determined how, for instance, make-up can best be applied to represent a cutaneous efflorescence or how the room has to be set up. Where necessary, the experiences from the SP trainer meetings can be further specified or adapted in the role descriptions.

**Translation: **Once the role descriptions have been finalised in the SP trainer meetings, the case is translated from the original German or French into the respective other language. To achieve as high a degree of uniformity as possible, the cases are each translated by the same two physicians into their native language.

**Quality assurance: **With this centrally coordinated, multistage process of exam development, involving content- and format-specific experts from all five faculties, it is ensured that the developed cases fulfil the format-specific requirements of an OSCE, pass through a national validation process, and can be employed at all faculties under standardised conditions. In terms of content validation, the task and function of the Review Board is of central importance. The national SP trainer meetings, in turn, have a decisive influence on the standardisation process.

#### Exam implementation

The FLE CS is conducted once a year at all five medical faculties on a decentralised basis. Depending on the number of candidates at the respective faculty, the examination is offered on two or three days. A different set of cases is used on each examination day; however, the examination sets are identical at all faculties on each respective day. The individual candidate completes 12 stations, each lasting for 13 minutes (with an additional two minutes rotation time between the stations and three 15-minute breaks). There is one physician-examiner in each room, and thus candidates are assessed by 12 different examiners in total. In addition to the case-specific ASM and generic CC criteria, the examiners assign two judgements on a global rating scale. These global judgements serve the purpose of calculating the pass mark.

**Quality assurance:** Detailed information (including video example) on the exam procedure is provided to the candidates in good time before the examination [http://www.bag.admin.ch/themen/berufe/07918/07919/ cited 14.12.13]. Clinicians who are assessing in the FLE CS for the first time are provided with orientation about the OSCE format at an information event and are instructed regarding how to handle the assessment grid. During and after the examination, the examiners have the possibility to give content-based feedback on the cases in general and on the assessment criteria specifically. Additionally, individual members of the WG CS visit the five faculties to get an idea of the exam administration at the different sites.

#### Exam evaluation

In a first step, all comments of the examiners are evaluated, and the difficulty and discrimination of the assessment criteria are calculated. On this basis, criteria which show content-related or formal deficiencies are eliminated. Following the elimination, the total score for a candidate at one station is calculated from the sum of the weighted checklist points of the ASM criteria added to the weighted score of the communication scale (total score=0.75*ASM+0.25*CC). The 12 stations all contribute equally to the total exam score. The candidates’ total exam score is calculated as the mean value across the 12 station scores. The candidates’ results are subsequently standardised in order to make the exam results comparable over the three days. In a next step, by means of the borderline regression method [12], [13], the pass mark per station is calculated. On this basis, the Board of Examiners determines the definitive pass mark. Table 1 (Tab. 1) shows the number of candidates separated according to groups with Swiss or non-European diploma and repeaters as well as the corresponding pass rates.

**Quality indices:** In table 2 (Tab. 2), some of the statistical quality indices of the FLE CS since 2011 are compiled. These results are in accordance with expectations insofar as the two exam formats MC and CS capture different aspects of medical competence but they (naturally) share the same basis. Moreover, the lower correlation of CC with MC (compared to ASM with MC) supports the construct validity of the examination.

**Quality assurance:** The data preparation and statistical analyses of the examination are undertaken according to established guidelines [14] centrally by one institution (Institute of Medical Education of the University of Bern). Additionally, the meticulous analysis of the examiners’ comments on the individual cases, which enables the elimination of flawed assessment criteria, contributes further to the quality of the examination. The low number of eliminated items is, in turn, a measure for the quality of the exam development process (in 2013, 22 of the 807 criteria (2.7%) were eliminated).

#### Central insights from the last three years

The iterative process with planning-implementation-reflection is considered by the WG CS to be an essential factor for the successful further development of the FLE CS. To illustrate this process using an example: The examiners’ feedback during the first two implementations indicated that dichotomous criteria are only partially suitable for assessing complex skills. Based on this experience, the members of the WG CS discussed possible alternative assessment criteria (planning). The goal was to adapt the assessment grid so that it can evaluate not only *whether* but also *how* a certain activity is performed. Based on these reflections, as well as consultation of the relevant literature, in the 2013 examination, rating scales were piloted (implementation). Results demonstrated that the correlations between scores on the global rating scale and scores achieved on the adapted checklist were higher at stations which contained these adapted rating scales compared to stations which contained only dichotomous criteria (reflection). The correlation of global judgement and total score is a criterion for the quality of an OSCE station. Accordingly, from 2014, where possible, adapted rating scales will be employed in the FLE CS for the assessment of complex skills.

Essential insights with regard to the individual steps of exam development, implementation and evaluation are described in the following. The centrally coordinated, collaborative process, with case development workshops, Review Board and national SP trainer meetings, and the incorporation of a large number of clinicians from all faculties and subject areas, is an essential factor of *exam development*. This consensus-oriented process makes a decisive contribution to the individual case as well as to the quality of the examination as a whole.

Since the first *exam implementation* in 2011, the examiners have evaluated the cases in terms of their medical content-based quality. Additionally, since 2013, the quality of SP performance has been captured by means of a standardised assessment grid. The use of this grid, with its explicit measurement criteria, which is given to the SPs in advance, has contributed to a further increase in the quality of SP performance as well as a further harmonisation between the individual sites.

In terms of the *exam evaluation*, the need emerged for additional quantitative parameters, which help to better judge the quality of the individual cases. With a set of statistical parameters described by Pell and others, different quality aspects of a station can be evaluated [15]. These parameters provide important information for the future process of case development, as they provide hints regarding an improvement of the assessment criteria, or where a standardisation is particularly demanding.

## Discussion

The current article presents the essential steps of exam development, implementation and evaluation, and provides central insights gained from the first three administrations of the Federal Licensing Examination Clinical Skills (FLE CS), from the perspective of the Working Group Clinical Skills.

A multistage procedure is recommended in international guidelines for the* development* of OSCE stations [16]. The centrally coordinated, collaborative-iterative process described here, with the participation of subject- and format-specific experts from all faculties, drives this principle forward and is an essential quality factor of the FLE CS. The *implementation* and *evaluation* of the FLE CS draws on the validated national Clinical Skills examinations from North America. For instance, the number of stations per candidate is identical to the United States Medical Licensing Examination Step 2 Clinical Skills [http://www.usmle.org/ cited 14.12.13], [17]. Additionally, the FLE CS has the following features in common with the Medical Council of Canada Qualifying Examination Part 2 [http://mcc.ca/examinations/mccqe-part-ii/ cited 14.12.13], [18]:

1. The candidates’ performance is assessed by clinicians of the respective teaching hospitals or universities;
2. The pass mark per station is calculated on the basis of expert assessments (global judgements);
3. The medical content and communicational aspects are added to form an overall score; and
4. The examination is offered in two languages.

Moreover, with regard to the pass rates, parallels between the US-American and the Swiss examination are apparent [19].

Among the central insights of the past years, one particular factor emerged: The consistent implementation of the principles of action research, in conjunction with the active participation of the many stakeholders, is essential for the successful further development of the FLE CS. On the whole, the FLE CS benefits from the experience of the OSCE examinations conducted at the individual faculties. However, this influence is not unidirectional. Rather, the faculty-based OSCEs have also benefited from the experiences with the FLE CS. Whereas, until recently, two to three persons at each faculty dealt locally with the OSCEs in isolation, since the introduction of the FLE CS, a Swiss-wide “community of practice” [20] has developed, which deals with the theme in a professional manner, cultivates a lively exchange, and strives to develop the structured clinical-practical examination further. The further development of this exam format, in particular for the use in licensing examinations, is not complete. Other institutions are also endeavouring to develop their national structured clinical-practical exams further [21].

At present, there are only a small number of countries worldwide with a national, structured clinical-practical examination [22]. Accordingly, only a small number of scientific projects have dealt with the development, implementation and evaluation of such examinations. The current article makes a first contribution to closing this gap. The essential steps and central insights documented here can be useful for interest groups, in particular with regard to the planning of national Clinical Skills examinations. However, it is also conceivable that the procedure we have described can be applied in ‘university consortia’, in order to offer a high-quality examination which exceeds the resources and expertise of one individual educational establishment.

The current work is limited to the perspective of the Working Group CS. It would be desirable in the future to also incorporate the experiences of candidates, lecturers and further stakeholders into the process. A further limitation of this work is that with such an extensive project, it is barely possible to provide a comprehensive and detailed description of all employed methods and the results obtained. 

## Conclusion

Based on the methodological foundation of action research, essential steps were presented which enable the successful implementation and further development of a national Clinical Skills examination, with which a candidate’s practical abilities can be assessed in a fair, reliable and valid manner. The processes and experiences documented in the current article can also be useful for other organisations planning a similar undertaking.

## Acknowledgements

A national Clinical Skills examination can only be accomplished through the commitment of countless persons. Our thanks go in particular to the case authors, members of the national Review Board, the Standardised Patient trainers, the Standardised Patients and the persons in charge at each site. We would also like to thank the clinicians for their work as examiners. Such an examination would also not be conceivable without the good collaboration and constant support of the Board of Examiners and the Federal Office of Public Health. Special thanks go also to Patrick Jucker-Kupper for the critical review of the manuscript.

## Competing interests

The authors declare that they have no competing interests.

## Figures and Tables

**Table 1:**
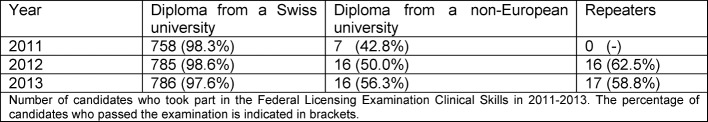
Number of candidates and pass rate for the Federal Licensing Examination Clinical Skills 2011-2013

**Table 2:**
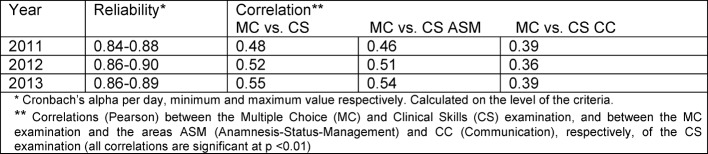
Statistical quality indices of the Federal Licensing Examination Clinical Skills 2011-2013
